# If so many are “few,” how few are “many”?

**DOI:** 10.3389/fpsyg.2015.00441

**Published:** 2015-04-17

**Authors:** Stefan Heim, Corey T. McMillan, Robin Clark, Stephanie Golob, Nam E. Min, Christopher Olm, John Powers, Murray Grossman

**Affiliations:** ^1^Department of Psychiatry, Psychotherapy, and Psychosomatics, Medical Faculty, RWTH AachenAachen, Germany; ^2^Research Centre Jülich, Institute of Neuroscience and Medicine (INM-1)Jülich, Germany; ^3^Jülich Aachen Research Alliance (JARA) – Translational Brain MedicineAachen, Germany; ^4^Department of Neurology, Frontotemporal Degeneration Center, University of Pennsylvania Perelman School of MedicinePhiladelphia, PA, USA; ^5^Department of Linguistics, University of PennsylvaniaPhiladelphia, PA, USA

**Keywords:** semantics, quantifiers, decision-making, numerosity, flexibility, learning

## Abstract

The scope of reference of a word's meaning can be highly variable. We present a novel paradigm to investigate the flexible interpretation of word meaning. We focus on quantifiers such as *“many”* or *“few,”* a class of words that depends on number knowledge but can be interpreted in a flexible manner. Healthy young adults performed a truth value judgment task on pictorial arrays of varying amounts of blue and yellow circles, deciding whether the sentence “Many/few of the circles are yellow” was an adequate description of the stimulus. The study consisted of two experiments, one focusing on *“many,”* one on *“few.”* Each experiment had three blocks. In a first “baseline” block, each individual's criterion for *“many”* and *“few”* was assessed. In a second “adaptation” block, subjects received feedback about their decisions that was different from their initial judgments in an effort to evaluate the flexibility of a subject's interpretation. A third “test” block assessed whether adaptation of quantifier meaning induced in block 2 then was generalized to alter a subject's baseline meaning for *“many”* and *“few.”* In Experiment 1, a proportion of yellow circles as small as 40% was reinforced as *“many”*; in Experiment 2, a proportion of yellow circles as large as 60% was reinforced as *“few.”* Subjects learned the new criterion for *“many”* in Experiment 1, which also affected their criterion for *“few”* although it had never been mentioned. Likewise, in Experiment 2, subjects changed their criterion for *“few,”* with a comparable effect on the criterion for *“many”* which was not mentioned. Thus, the meaning of relational quantifiers like “many” and *“few”* is flexible and can be adapted. Most importantly, adapting the criterion for one quantifier (e.g., *“many”*) also appeared to affect the reciprocal quantifier (in this case, *“few”*). Implications of this result for psychological interventions and for investigations of the neurobiology of the language-number interface are discussed.

## Introduction

Humans are very efficient at processing quantities. Assessing and evaluating the number of desirable or potentially dangerous entities—chunks of food, precious artifacts, or deadly animals—happens in fractions of seconds. Importantly, we are also able to share these rapid impressions with others: Concrete quantities or abstract symbolic representations can be translated into number words (cf. Dehaene et al., 2003) or, similarly, other number-describing expressions, so-called quantifiers (e.g., *“many,”* “*at least five*,” etc.).

Quantifiers can take the form of different semantic classes. On the one hand, there are cardinal quantifiers (“*three* bottles”) which contain an explicit numeric expression (in our example: three). There are also other classes of quantifiers that refer to quantity with an implicit notion of quantity that do not refer to an exact number or numeric interval. For instance, Aristotelian quantifiers (comprising existential quantifiers such as “*all* X are Y” and logical quantifiers like e.g., “*some* X are Y”) or majority quantifiers (“*at least half* of the X are Y”) refer to quantities in the environment in the absence of an explicitly defined number. Items in the environment may be defined by the total size of a set: If there are 12 oranges, for example, “*at least half* of the oranges” means more than 6. However, in other cases, the criterion may be defined more vaguely. For instance, “*much* chocolate” may mean one bar or five bars, depending on the individual's preference. Likewise, it may depend on the semantic context: “*many* lions” could be five, but “*many* ants” probably means hundreds or thousands. We focus our study on quantifiers because we can easily and precisely measure the meaning of this class of word, even one that does not explicitly mention a number. This is because the meaning of a quantifier is derived in part from number knowledge—to varying degrees depending on the exact nature of the quantifier.

Majority quantifiers refer to a subset of items that require a computation based on an understanding of the entire set of items and thus tend to be more difficult than cardinal quantifiers where an explicit number is mentioned. For instance, children use number expressions much earlier, and far more correctly, than quantifiers (Hurewitz et al., 2007; Sullivan and Barner, 2011). In particular, the appropriate use of quantifiers is not always easy. Consider the sentence “All shirts are blue.” If this sentence is true, then the sentence “Some shirts are blue” is, logically, also true (cf. Geurts, 2003). This phenomenon is called scalar implicature, indicating that if the truth value for a set of elements is 1, then it is also 1 for any given subset. However, as Grice (1967) pointed out, this is not how we use quantifier expressions in every-day conversation. Rather, we use “conversational implicatures,” trying to be as relevant as possible (for a discussion see Haugh, 2002). In the given example, this would mean that we distinguish between *“some”* and *“all”* such that *“some”* means “only *some*, not *all*.”

This Gricean use of quantifiers can be demonstrated empirically in the laboratory: Different quantifiers are used to refer to different ranges of numerosities (e.g., Oaksford et al., 2002). For a given set of objects, “none” refers to the lower end of the scale, followed by *“few,” “some,” “some not,” “most,”* and *“all”* in ascending order. This order was reliably observed with different tasks, indicating that a given quantifier is most appropriate, and thus most informative, for a certain fraction of the overall numerosity.

However, the distinction between scopes of quantifiers is not completely clear-cut. Rather, scopes of quantifiers tend to overlap partially (e.g., Holyoak and Glass, 1978; Oaksford et al., 2002). This may lead to situations in which two quantifiers are (nearly) equally appropriate for the description of a given amount of entities. For instance, in the Oaksford et al. (2002) study, participants were presented with images containing varying amounts of black or white squares, together with a statement like “*Most* of the squares are black.” Their task was to decide whether the statement was an appropriate description of the visual scenario. When there were about 18% of black squares on the screen, subjects accepted the statement “*Few* of the squares are black” in about 75% of the cases. Interestingly, when presented with the same scenario but the sentence “Some of the squares are black,” they would accept the statement in exactly the same proportion of cases. Compatible results were found in a memory experiment by Holyoak and Glass (1978) who demonstrated that quantifiers like *“many”* and *“a few”* might be confused in memory in some proportion of cases, the chance being higher as the quantities and the referring quantifiers became more similar. To summarize, the evidence suggests that the selection of quantifiers for every-day use seems to follow the Gricean principle of relevance, maximizing the information expressed by this quantifier. Such specific selection is possible because each quantifier has a particular scope, i.e., it refers to a certain interval of probabilities distributed around one criterion value which best represents the semantics of the quantifier. These scopes overlap at least in part.

Given this overlap of scopes and the fact that quantifiers may be exchanged under some circumstances, a question arises regarding the invariance of the criteria to which quantifiers refer in an individual. Given the close relationship between processing of numbers and numerosities on the one hand and quantifiers on the other hand, findings from studies of number processing may shed some light on this question. Both behavioral and neurophysiological studies (e.g., Nieder and Miller, 2003; Piazza and Izard, 2009) indicate that processing of numerosities is not an all-or-nothing phenomenon. Rather, even if a presented number of elements does not exactly match a target numerosity, neural assemblies may also fire to these if they are close enough to the target numerosity, resulting in the subject to show overt behavioral responses to this slightly deviant numerosity. For instance, Piazza and Izard (2009) summarize a series of studies in monkeys and human subjects who performed same-different judgment tasks on stimuli with dot patterns. Intracortical recordings in parietal neurons in monkeys showed the firing pattern described above, which nicely corresponded to the distribution of the percentage of “same” responses in the monkeys. In human subjects, fMRI was used instead of intracortical recordings, yielding again a nice match between button press responses and the shape of the BOLD response in the left parietal cortex around the intraparietal sulcus.

Most interestingly, these behavioral responses are not invariant. In his seminal work, Helson (1948) demonstrated that the repeated exposure to a certain stimulus intensity, or numerosity, may result in a change of what he called “adaptation level.” Responses to the former target intensities, or numerosities, are thus altered, leading to a so-called peak shift in the response curves. The maximum probability of a YES response is moved toward a new criterion.

Following the argument above that the processing of quantifiers and numbers bears some similarities, one might assume that such a peak shift phenomenon can occur not only for number processing, but also for quantifier processing. In other words, some external learning influence is likely to change the internal criterion to which a quantifier refers, resulting in a shift of the scope of this quantifier. It is yet an open question whether such a phenomenon may occur. If so, another question follows: Does the scope of this one quantifier shift in isolation, or does it also affect the scope of other quantifiers? In particular, a change in the criterion for “many” affect the mental representation of the related quantifier “few?” If true, this would provide some support for the claim that quantifiers like “many” and “few” are linked in their representations in semantic memory even though these terms refer to different quantities (cf. Routh, 1994).

The present study was thus conducted in order to address these two questions. For this purpose, we used the semantic truth value judgment (STVJ) task by Heim et al. (2012) and combined it with explicit feedback trials as e.g., used by McMillan et al. (2012). As a result, we were able to assess the initial criteria to which the quantifiers *“many”* and *“few”* refer, to train subjects to change their internal criteria for “many-ness” (Experiment 1) or “few-ness” (Experiment 2) independently from each other, and to test whether explicitly altering the one criterion (e.g., for *“many”*) would also affect criterion for the other, untrained quantifier (in this case, for *“few”*). We used the quantifiers *“many”* and *“few”* because they are familiar, they do not differ in processing requirements (see above), and at the same time they are sufficiently distant from each other (Routh, 1994) to be not mistaken too easily.

## Experiment 1

The purpose of Experiment 1 was to assess the subjects' initial criteria for calling a certain amount of circles of a given color *“many”* or *“few”* (Block 1), then to shift the criterion for *“many”* explicitly to a lower proportion of circles (Block 2), and finally to test whether this shift also affected the criterion for *“few”* even though the criterion for this quantifier was not mentioned (Block 3). All subjects participated in an informed consent procedure that was approved by an Institutional Review Board convened at the University of Pennsylvania.

### Methods

#### Participants

Twenty-one healthy volunteers (mean age 22.7 years, range 19–29 years; 13 women; average amount of education: 15.8 years) participated in this study. They were recruited at the University of Pennsylvania and received $10 for their participation.

#### Procedure

Subjects were presented with pictures showing isoluminant blue and yellow circles on a gray background. The stimuli were taken from the study by Heim et al. (2012). Each stimulus item included a picture containing 50 circles, with proportions of yellow circles ranging from 20/30/40/50/60/70% of the total number of circles. The remaining circles were blue. There were six different stimuli for each proportion. In order to minimize learning effects due to repetition of stimuli, each stimulus was presented in three different rotations (0°, 90°, 180°), a procedure that had previously been shown to reduce repetition memory effects for stimuli (Lassaline and Logan, 1993).

Each picture was presented together with the sentence “*Many* of the circles are yellow” or “*Few* of the circles are yellow.” The subjects performed a truth value judgment task, indicating whether they thought the sentence adequately described the picture or not by pressing the left or right response button^1^.

**Figure 1:**
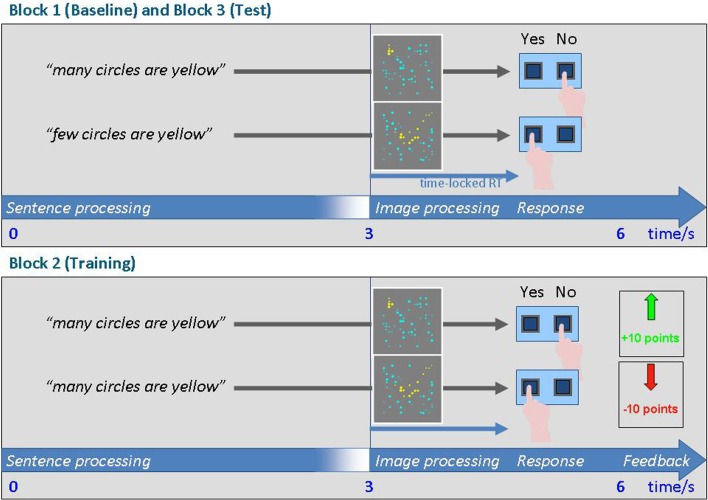
**Schematic of the trials in blocks 1 and 3 (top) and in the adaptation block 2 (bottom)**.

The experiment consisted of three blocks (cf. Figure 1). The first block served as a baseline block, in which the subjects' internal criteria for *“many”* and *“few”* were assessed. To this end, subjects performed the STVJ task in a total of 378 trials counterbalanced for “few” and “many” trials along with the proportion of yellow circles. After this baseline block, an adaptation block followed in which subjects received feedback for their response. In this block, we provided feedback and only stimuli with the quantifier *“many”* were presented. Positive feedback was given if they responded YES to pictures containing 40% or more of yellow circles, or NO to pictures with less than 40% of yellow circles. Negative feedback was given if they responded NO to pictures with 40% or more, or YES to less than 40% yellow circles. Based on previous data (Heim et al., 2012), this feedback would effectively move the internal criterion from a presumed baseline of 50-60% down to 40%. For this adaptation block for *“many,”* proportions of 20–70% yellow circles were used.

The adaptation block consisted of 162 trials, which were organized to form three sub-blocks with equal amounts of trials of each proportion. Sub-dividing the adaptation block into these three homogeneous sub-blocks allowed us to assess the learning curve over the adaptation block with more precision by allowing us to analyze each sub-block independently. Proportions were distributed equally over the first, second, and third part of the adaptation phase, thus creating three parallel sub-blocks of 54 trials each (9 trials for each of the 6 proportions).

After the adaptation block, the test block was administered. This was identical to the baseline block, i.e., sentences containing *“many”* or *“few”* were presented, and no feedback was given. The test block was run to assess whether and how the internal criterion for *“many”* had been changed during the explicit training block for *“many,”* and whether the criterion for *“few”* had been altered in parallel even though *“few”* had never been presented in the adaptation block.

Prior to the real experiment, all subjects were familiarized with the stimuli and task in two short blocks, one without feedback that resembled the baseline or test phase, and one with feedback as in the adaptation phase.

The timing of a baseline/test trial is illustrated in Figure 1A. First, a written sentence (“Many of the circles are yellow” or “Few of the circles are yellow”) was shown in the upper third of the screen for 3 s. The sentence stayed on for another 1.5 s while the stimulus picture containing yellow and blue circles appeared at the center of the screen for the same amount of time. The words “YES” and “NO” were written in the lower third of the screen to indicate which button to push for which decision. Finally, the screen went blank for another 1.5 s before the next trial began. Responses were recorded from the onset of the picture and through the entire blank period.

The trials in the adaptation phase are illustrated in Figure 1B and were similar to those in the baseline/test phase, with the exception that feedback was given in these trials. If the subjects responded correctly, they received 10 points which were added to their score. False responses were penalized by deducting 10 points from their score. The feedback screen consisted either of a green arrow pointing upward and the information “+10” in green font, or a downward red arrow and the text “−10” in red font. The initial score was “+100.” The actual score was always presented in the middle of the very upper part of the screen in black font. The adaptation phase consisted of 162 trials. Proportions were distributed equally over the first, second, and third portions of the adaptation phase, thus creating three parallel sub-blocks of 54 trials each (9 trials for each of the 6 proportions). These sub-blocks could then be compared in order to test how quickly the subjects learned.

#### Data analysis

Because of the balanced assignment of YES/NO responses to the left/right response button, the subjects' button press responses were first recoded into “1” (YES) or “0” (NO) to make data sets comparable. Next, these acceptability ratings were aggregated per subject, experimental block, quantifier, and proportion of circles of the target color. Consequently, a 2 × 2 × 6 ANOVA with factors BLOCK (baseline/test), QUANTIFIER (many/few), and PROPORTION (20/30/40/50/60/70) was run to test for differential learning effects for the trained vs. untrained quantifier at trained vs. untrained proportions. Subsequently, planned contrasts (paired *t*-tests) were computed to compare the change in acceptability ratings at the critical proportion “40%” for the trained and the untrained quantifier. In a second analysis, we also tested how quickly the subjects learned during the adaptation phase. To this end, data of the adaptation block were collapsed over subject, sub-block, quantifier, and proportion. Paired *t*-tests (sub-blocks 1 vs. 2, 2 vs. 3, and 1 vs. 3) were run to compare the acceptability ratings at the critical proportion “40%.” We used one-tailed *t*-tests because of our a priori predictions.

### Results

#### Adaptation effects

**Figure 2:**
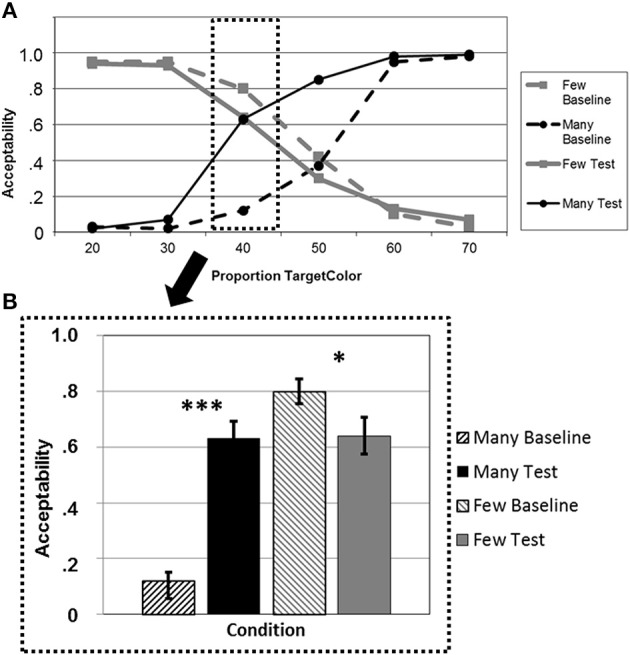
**(A)**. Average acceptability ratings for a given proportion of circles of the mentioned color, plotted separately for the quantifiers *“many”* (black lines) and *“few”* (gray lines) in the baseline blocks (dashed lines) and the test blocks after adaptation (solid lines). **(B)** Average acceptability ratings for a critical proportion of circles of the mentioned color, plotted separately for *“many”* (black bars) and *“few”* (gray bars) in the baseline blocks (dashed bars) and the test blocks after adaptation (solid bars). ^*^p < 0.05, ^***^p < 0.001.

The 2 × 2 × 6 ANOVA yielded significant main effects for BLOCK [*F*_(1, 20)_ = 28.69; *p* < 0.001] and PROPORTION [*F*_(5, 16)_ = 5.50; *p* = 0.004] but not for QUANTIFIER [*F*_(1, 20)_ < 1]. Moreover, all two- and three-way interactions were significant [QUANTIFIER × BLOCK: *F*_(1, 20)_ = 34.39; *p* < 0.001; QUANTIFIER × PROPORTION: *F*_(5, 16)_ = 1362.62; *p* < 0.001; BLOCK × PROPORTION: *F*_(5, 16)_ = 4.24; *p* = 0.012; BLOCK × QUANTIFIER × PROPORTION: *F*_(5, 16)_ = 9.73; *p* < 0.001]. These effects demonstrate a strong adaptation effect for the trained quantifier *“many,”* and most importantly, we also observed a transfer effect for the not trained quantifier *“few”* (Figure 2A).

Testing the adaptation effect for *“many”* and *“few”* at the critical proportion “40%” illustrates this overall effect (Figure 2B). We found a highly significant increase in acceptability for *“many”* [*t*_(20)_ = −7.79; *p* < 0.001], and a decrease for *“few”* [*t*_(20)_ = 2.58; *p* = 0.018] (one-tailed *t*-tests, *p*-Bonferroni-corrected).

#### Learning during the adaptation phase

**Figure 3:**
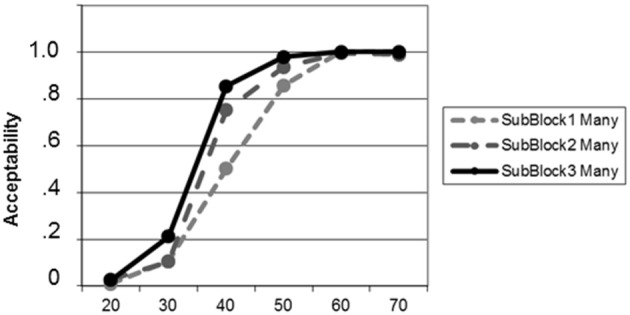
**Learning curves for the new meaning of *“many”* during the adaptation phase (block 2) as a function of percent circles of the target color, plotted separately for each of the three sub-blocks**. The learning effect at the trained proportion “40” is visible.

Testing the learning effect for *“many”* in block 2 yielded on-going adaptation (Figure 3). This effect was significant from sub-block 1 to sub-block 3 [*t*_(20)_ = −7.75; *p* < 0.001] as a result of increases from sub-block 1 to sub-block 2 [*t*_(20)_ = −5.03; *p* < 0.001] and from sub-block 2 to sub-block 3 [*t*_(20)_ = −1.83; *p* = 0.042; all tests one-tailed, uncorrected for multiple comparisons].

### Discussion

In this study we investigated whether individuals can flexibly adapt their interpretation of quantifier meaning. Moreover, we tested whether such change could affect the criterion of an untrained quantifier as well, thus indicating a shift in the entire semantic reference frame for numerosities. For both research questions we obtained positive evidence. Subjects were able to shift their internal criterion for *“many”* toward the new reference criterion of “40%” following explicit training. A closer examination of the dynamics of this change (Figure 3) revealed that learning actually happened quite rapidly, with substantial changes in the acceptability ratings from the first third to the second third of the adaptation phase. Most interestingly, the comparison between baseline and test phase, which were absolutely identical with respect to quantifiers, stimuli, and their pairings, revealed that the internal criterion for the second quantifier (*“few”*) had also shifted although it had not been mentioned in the adaptation phase.

These findings have interesting implications. They suggest that contextual influence such as explicit reinforcement can be a driving force to shift the quantifier's scope (cf. Price et al., 2014 for an extensive discussion of feedback mechanisms during the estimation of quantities). Moreover, there is a change in the reference of the related quantifier “few,” even though this was not explicitly mentioned during the adaptation phase. This suggests that subjects' learning was not limited to the superficial mapping of a word to a stimulus, but that the concept underlying this continuum of quantifier word meaning was changed. The data are consistent with the interpretation that the contextual appropriateness of a quantifier (such as the untrained *“few”* in the present study) depends on its relative position in the continuum of reference criteria: If a proportion of 40% circles of a given color is best described as *“many,”* the initial tendency of a subject to call 40% *“few”* cannot be maintained. If it were maintained, the Gricean principle of maximized information would be violated because two different quantifiers that are partly exclusive and that are on different positions on the continuum of reference criteria (Oaksford et al., 2002) would describe the same semantic reality.

Yet, the interpretation of this data set is limited in two ways. First, a direct shift for the trained quantifier and a transfer for the untrained quantifier were only observed for a decrease of the criterion (from 50 to 40%), i.e., leftwards on the continuum of reference criteria. Second, the trained quantifier *“many”* was positive. From earlier studies (e.g., Heim et al., 2012) it is known that positive quantifiers such as *“many,”* “most,” or “more than half” are easier to process than their negative counterparts, e.g., *“few”* or “less than half.” In line with cognitive load theories (e.g., Bannert, 2002) one might assume that the learning transfer observed in Experiment 1 would diminish if the cognitive load associated with the quantifier would be higher, as it would be in the case of the negative quantifier *“few.”* This is because there may be reduced potential mental flexibility associated with a word requiring increased cognitive load during processing. Therefore, we conducted Experiment 2 in which the setting was changed accordingly: Here we explicitly trained *“few,”* and then examined whether a transfer effect would be observed for *“many.”* Second, since the acceptability ratings for *“few”* in the baseline block of Experiment 1 indicate ceiling effects at low proportions, the criterion shift was now rightwards, i.e., toward the higher reference criteria 50 and 60%.

## Experiment 2

Thus, the questions in Experiment 2 were the following. (1) Can the criterion of a negative quantifier like *“few”* (the scope of which is on the mental number line below the criterion, e.g., “*less than half*” or “*fewer than five,”* not above as in the case of positive quantifiers such as “*many”* or “*more than half”*) be changed in a manner that is analogous to that of *“many”* in Experiment 1? (2) Is there a generalization of this learning effect to the untrained quantifier *“many”*? Again, all subjects participated in an informed consent procedure that was approved by an Institutional Review Board convened at the University of Pennsylvania.

### Methods

The same subjects as in Experiment 1 completed another experiment that was almost identical to Experiment 1. The only difference was the following: In block 2 of Experiment 2, the subjects were now trained to call proportions of 50% and 60% of circles *“few.”* YES responses were encouraged for 60% or less yellow circles, while NO responses were reinforced for proportions above 60% yellow circles. In Experiment 2, the proportion ranged between 30 and 80% of circles of the target color. The order of Experiments 1 and 2 were counterbalanced within a single experimental session such that potential carry-over effects from one experiment to the other could be controlled (10 subjects started with Experiment 1, 11 started with Experiment 2). Analyses were analogous to those in Experiment 1.

### Results

#### Adaptation effect

**Figure 4:**
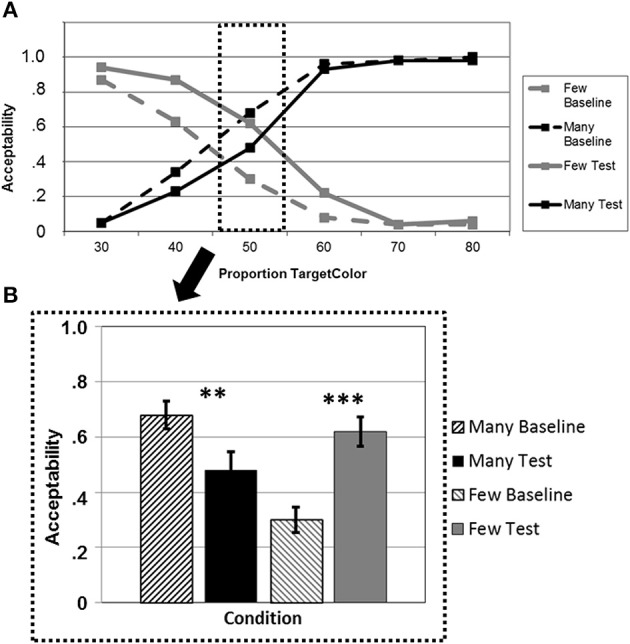
**(A)** Average acceptability ratings for a given proportion of circles of the mentioned color, plotted separately for the quantifiers *“many”* (black lines) and *“few”* (gray lines) in the baseline blocks (dashed lines) and the test blocks after adaptation (solid lines). **(B)** Average acceptability ratings for a critical proportion of circles of the mentioned color, plotted separately for *“many”* (black bars) and *“few”* (gray bars) in the baseline blocks (dashed bars) and the test blocks after adaptation (solid bars). ^**^p < 0.01, ^***^p < 0.001.

The 2 × 2 × 6 ANOVA yielded significant main effects for BLOCK [*F*_(1, 20)_ = 5.82; *p* = 0.026] and QUANTIFIER [*F*_(5, 16)_ = 78.67; *p* < 0.001] and a trend for PROPORTION [*F*_(1, 20)_ = 2.71; *p* = 0.059]. Moreover, the following interaction terms were significant (QUANTIFIER × BLOCK: *F*_(1, 20)_ = 25.61; *p* < 0.001; QUANTIFIER × PROPORTION: *F*_(5, 16)_ = 763.71; *p* < 0.001; BLOCK × QUANTIFIER × PROPORTION: *F*_(5, 16)_ = 6.92; *p* = 0.001]. The interaction term for BLOCK × PROPORTION was marginally significant [*F*_(5, 16)_ = 2.43; *p* = 0.081]. These effects describe a strong adaptation effect for the trained quantifier *“few”* and a weaker transfer effect for the not trained quantifier *“many”* (Figure 4A).

Testing the learning/transfer effect for *“many”* and *“few”* at the critical proportion “50%” pinpoints this overall effect (Figure 4B), showing a highly significant increase in acceptability for *“few”* [*t*_(20)_ = −5.49; *p* < 0.001] and a significant decrease for *“many”* [*t*_(20)_ = 3.10; *p* = 0.006] (one-tailed *t*-tests, *p*-Bonferroni-corrected). At proportion “60%,” there was again a significant learning effect for *“few”* [*t*_(20)_ = −2.90; *p* = 0.009] but no effect for *“many”* [*t*_(20)_ = 0.84; *p* = 0.411].

#### Learning during the adaptation phase

**Figure 5:**
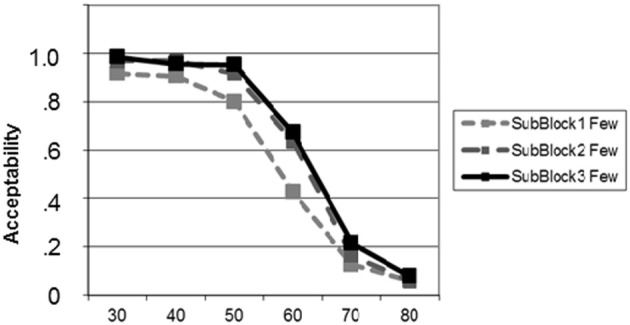
**Learning curves for the new meaning of *“few”* during the adaptation phase (block 2), plotted separately for each of the three sub-blocks**. Learning effects for proportions 50 and 60 are visible.

Testing the learning effect for *“few”* in the adaptation block yielded on-going learning (Figure 5). This effect was significant from sub-block 1 to sub-block 3 [*t*_(20)_ = −3.57; *p* = 0.001] as a result of a significant increase from sub-block 1 to sub-block 2 [*t*_(20)_ = −2.55; *p* = 0.005] and a strong trend from sub-block 2 to sub-block 3 [*t*_(20)_ = −1.67; *p* = 0.056; all tests one-tailed, uncorrected for multiple comparisons].

#### Stronger adaptation effect for “many” (Experiment 1) than for “few” (Experiment 2)

Next, in order to test whether the direct adaptation effect for *“few”* observed in Experiment 2 was equal in size to that for *“many”* in Experiment 1, we tested across experiments for the interaction of BLOCK and trained QUANTIFIER at the respective critical proportions “40%” (for Experiment 1) and “50%” (for Experiment 2). This interaction effect proved to be significant [*F*_(1, 20)_ = 4.44; *p* = 0.048], indicating that the adaptation effect for *“many”* was larger in size than that for *“few.”*

#### No effect of the order of Experiments 1 and 2

Finally, in order to test whether the order in which the subjects underwent the two experiments had any systematic influence on the data pattern, we re-ran this analysis adding ORDER as a between-subject factor. Neither the main effect of ORDER [*F*_(1, 19)_ = 1.10; *p* = 0.308) nor any 2-way or 3-way interaction with ORDER was significant (all *p* > 0.05).

### Discussion

In Experiment 2, we tested whether the pattern of results from Experiment 1 would be mirrored in adaptation effects for the negative quantifier *“few”* with a generalization to the untrained quantifier *“many.”* The findings were straight-forward. There was a clear learning effect for few at both trained proportions “50%” and “60%” as well as a transfer to the criterion for *“many”* at “50%.” Moreover, the in-depth analysis of the learning curves in the three parts of the adaptation block revealed again relatively rapid learning of the new criterion from the first-third to the second-third of the adaptation phase.

These data corroborate the initial findings and conclusions, i.e., that a change in meaning of a quantifier may be contextually induced and that this change in meaning may also affect related quantifiers even if they are not exact opposites or antonyms. The fact that the same subjects underwent both experiments precluded problems of between-group comparisons; the fact that the order of experiments was counterbalanced over subject ensured that the adaptation and transfer effects observed in Experiment 2 were not just a resetting to the subjects' original criteria temporally shifted for the duration of Experiment 1. Still, we found that the training for *“many”* elicited a stronger adaptation effect than that for *“few.”* The implications of the overall pattern of results will now be discussed in more detail.

## General discussion

In this paper we reported two experiments that tested whether the meaning of a quantifier such as *“many”* or *“few”* can be altered, and whether this would affect the meaning of another, untrained quantifier as well. The findings were straight-forward, with effects for explicit training as well as transfer effects to the untrained quantifier for both quantifiers. These findings emphasize the flexible representations of conceptual knowledge.

These data have several implications for the field of quantification and reasoning. For one, they suggest a certain flexibility of our interpretation of quantifiers that depends in part on the context. As in the introductory example, *“many”* can imply different amounts of e.g., animals depending on whether they are rare (“*many* lions” = 10) or not (“*many* ants” = 10,000), i.e., the semantic context in which it is used. As Sanford and Moxey (e.g., in their 2003 paper) point out, quantifier use may reflect the individual perspective on, or interpretation of, a quantity rather than an inflexible, fixed characterization of its magnitude. This is in line with the finding that quantifier scopes partly overlap, as could be shown by Holyoak and Glass (1978). These authors asked subjects to provide not only their first choice of an appropriate quantifier but also their second choice. They found for this latter task preference for quantifiers with closest distance on the continuum (all—many—some—a few—none).

However, the notion of a flexible, partly subjective perspective on quantifier meaning may not be true for quantifiers in general. According to Routh (1994), one way of grouping quantifiers is with respect to the degree of variability of the criterion they refer to. Routh distinguished between “fuzzier quantifiers” such as *“few,” “several,”* or *“many”* that are determined in part by context, Aristotelean or logical quantifiers that are “more precise” such as *“all,” “none,”* or *“each,”* and parity or majority quantifiers that are “comparative” like *“more”* or *“fewer.”* This distinction is based in part on the flexibility of the criteria for the referents of a given quantifier. Whereas “many donuts” may be two for person A and five for person B, comparative quantifiers imply at least the imparity of sets, i.e., ordinal information about which set is bigger. Most objective are quantifiers for which the criterion is explicitly or implicitly named, as in the case of “at least 13” or “more than half” (the latter referring to an amount of more than 50% of the total set)—objective in the sense that they refer to an explicitly stated criterion or degree. Routh (1994) presented evidence from clustering algorithms and multidimensional scaling that this classification of quantifiers has psychological and empirical reality and is thus effective in communication. Given this distinction, one could formulate the hypothesis that the observed changes for quantifier semantics in the present study might generalize to other “fuzzy” quantifiers such as “some,” “a few,” “several,” “quite a few,” etc.

Interestingly, within the class of “fuzzy quantifiers” like the majority quantifiers *“many”* and *“few”* tested in the present study, there seem to be differences between individual quantifiers. Whereas we did observe adaptation of the meaning of both quantifiers, we also found that this adaptation effect was even more pronounced for *“many”* than for *“few.”* This result corresponds to earlier reports in the literature (e.g., Routh, 1994; Geurts, 2003; Heim et al., 2012; Shikhare et al., 2015) that quantifiers with negative polarity are more difficult to process. There are various accounts for this effect. One stresses that the positive quantifiers are (unmarked) defaults whereas negative quantifiers are marked (Clark, 1969; Clark and Chase, 1972). Alternatively, it has been proposed that negative quantifiers refer more or less precisely to the complement of the set denoted by a positive quantifier. Thus, in order to obtain the correct representation of a negative quantifier, the expression about the original set has to be negated, implying extra processing costs^2^. Much more work is required here. Still, in the context of the present study, it could well be that negation or markedness pose greater cognitive demands on the comparison of a negative polarity quantifier like *“few,”* resulting in gradually smaller flexibility and thus a smaller adaptation effect.

If the meaning of a “fuzzy quantifier” can be changed, the question arises in how far such flexibility can also be found for quantifiers that are less fuzzy, i.e., that have fixed criteria. While the current study suggests that quantity can modulate quantifier interpretation, there is also additional evidence suggesting that object size, or mass, can modulate quantifier interpretation (McMillan et al., 2013). The use of quantifiers with different degrees of fuzziness in one experiment may restrict the flexibility of e.g., many and few that was observed here. Alternatively, it could also be the case that shifts along the continuum of criteria might even affect quantifiers with a fixed criterion: For instance, in the case of “at least X,” subjects might be more inclined to accept such a statement when the distance of the actual numerosity to the criterion increases (cf. Shikhare et al., 2015, for first results on distance effects related to “at least” and “at most”).

Our findings address the question of the flexibility of word meaning, such as deciding when the diameter of a cup becomes broad enough to be called a bowl. This has been a challenging issue to address because of the difficulty quantifying the associated object concepts. Here we addressed this issue by examining quantifiers, a class of words that is based in part on number knowledge. We found that word meaning is highly flexible. It is trivial to re-label a concept, as in our explicit training paradigm. However, we also found a shift in the meaning of untrained words. This suggests a modification of word meaning at a deeper, conceptual level. The mental flexibility allowing the development of a modified meaning was not transient and determined by the experimental paradigm, but appeared to be maintained throughout the test block. Thus, in contrast to models of word meaning that depend entirely on a set of fixed features (Locke) or reference to an expert (Goodman), our findings are compatible with the notion that a component of word meaning is associated in part with a central tendency (Wittgenstein) that is somewhat flexible in the reference of a word.

Another question that needs to be addressed in future research is how long the criterion shift effect induced in the adaptation phase actually lasts, i.e., how long it takes for the learning curve to go back to baseline level. Any considerations of how to make use of the observed effect in therapeutic settings where magnitudes or amounts are being misevaluated by patients (as in additive disorders or body schema disorders) will depend on its natural decay. This decay may in part depend on working memory processes which have been discussed as essential for quantifier processing (McMillan et al., 2005; Zajenowski et al., 2014).

The present findings and the paradigm used to produce them may be helpful in order to resolve a longer-standing debate about the nature of the brain network underlying quantifier processing. McMillan et al. (2005) found increasing involvement of the left inferior frontal cortex in quantifiers with increasing processing demands (*“at least 3,” “all,” “some”* vs. *“less than half,” “odd,” “even”*) and related this effect to aspects of working memory (see Wei et al., 2014, for comparable findings in Chinese). More recently, Heim et al. (2012) reported two fronto-parietal networks for quantifier processing in the left hemisphere that were neighboring but hardly overlapping. One of these networks was related to the initial estimation of numerosities, the other to the comparison with a criterion. Interestingly, only the frontal component also showed systematic effects of processing quantifier meaning, suggesting a functional segregation between the frontal and the parietal components of the two networks. The paradigm presented here may be useful to get deeper insights into the role of the frontal component in order to understand in how far it is involved in the processing of change of meaning during the adaptation phase and/or maintenance of the newly learned meaning in working memory and its decay. Thus, subsequent work both with functional neuroimaging and also with patients with frontal or parietal neurodegeneration is encouraged in order to identify the neural basis of quantifier processing from the initial percept to the final semantic evaluation. Some first steps into that direction have been done by McMillan et al. (2013) and by Morgan et al. (2011) in studies of patients with fronto-temporal and/or posterior cortical disease. In accordance with Dehaene's triple-code model (Dehaene et al., 2003) it was again atrophy in the frontal cortex that had a severe effect on the verbal code of numerosities. It is yet an open question whether it is the dysfunction of parts of the frontal cortex itself, or rather dynamic diaschisis (i.e., alterations in the entire network of frontal and parietal areas, as could be supposed based on Troiani et al., 2009) that thus affects the neurobiology of the language-number interface in the human brain.

## Conclusion

The present study investigated quantifier processing in a setting in which their meaning could be experimentally altered for the course of the experiment. This novel paradigm yielded results suggesting that, in order to be most highly informative, a change in meaning in one quantifier generalizes also to that of other quantifiers, i.e., to a larger referential frame. Future studies are required to test the usefulness and limits of this paradigm behaviorally and with neuroimaging.

### Conflict of interest statement

The authors declare that the research was conducted in the absence of any commercial or financial relationships that could be construed as a potential conflict of interest.

## Footnotes

^1^The assignment of the YES and NO responses to the one or other button were kept constant for each single subject for the duration of the entire experiment. Across subjects, the assignment of YES or NO to the left or right button was balanced. Thus, 50% of the subjects pressed the left button for YES and the right for NO and vice versa for the other 50%.

^2^Note, however, that “few” is not exactly equivalent to “not many.” This is due to the fact that these two quantifiers do not refer to the same reference, or criterion, whatever its value may be in a given context and for a given individual. In the example of peanuts, it is well possible that you neither eat few peanuts (i.e., a handful) nor many (i.e., the whole package), but just some intermediate amount. The situation might be different for quantifiers that are exact opposites not by virtue of explicit negation (“more than half”/“not more than half”) but of semantic polarity (“more than half”/“less than half”).
